# Dexmedetomidine post-conditioning attenuates cerebral ischemia following asphyxia cardiac arrest through down-regulation of apoptosis and neuroinflammation in rats

**DOI:** 10.1186/s12871-021-01394-7

**Published:** 2021-06-28

**Authors:** Guangqian Li, LeiQian, Pan Gu, Dan Fan

**Affiliations:** 1 School of Medicine, Universityof Electronic Science and Technology of China, Chengdu, China; 2 Department of Anesthesiology, Sichuan Academy of Medical Sciences and Sichuan Provincial People’s Hospital, No. 32 West Second Section, First RingRoad, Chengdu, 610072 Sichuan China

**Keywords:** Dexmedetomidine, Post-conditioning, Cerebral ischemia, Asphyxiacardiac arrest, Apoptosis, Neuroinflammation

## Abstract

**Background:**

Neuroprotection strategies after cardiac arrest (CA)/cardiopulmonary resuscitation (CPR) remain key areas of basic and clinical research. This study was designed to investigate the neuroprotective effects of dexmedetomidine following resuscitation and potential mechanisms.

**Methods:**

Anesthetized rats underwent 6-min asphyxia-based cardiac arrest and resuscitation, after which the experimental group received a single intravenous dose of dexmedetomidine (25 μg/kg). Neurological outcomes and ataxia were assessed after the return of spontaneous circulation. The serum levels and brain expression of inflammation markers was examined, and apoptotic cells were quantified by TUNEL staining.

**Results:**

Neuroprotection was enhanced by dexmedetomidine post-conditioning after the return of spontaneous circulation. This enhancement was characterized by the promotion of neurological function scores and coordination. In addition, dexmedetomidine post-conditioning attenuated the serum levels of the pro-inflammatory cytokine tumor necrosis factor (TNF)-α at 2 h, as well as interleukin IL-1β at 2, 24, and 48 h. TUNEL staining showed that the number of apoptotic cells in the dexmedetomidine post-conditioning group was significantly reduced compared with the control group. Further western blot analysis indicated that dexmedetomidine markedly reduced the levels of caspase-3 and nuclear factor-kappa B (NF-κB) in the brain.

**Conclusions:**

Dexmedetomidine post-conditioning had a neuroprotective effect against cerebral injury following asphyxia-induced cardiac arrest. The mechanism was associated with the downregulation of apoptosis and neuroinflammation.

## Background

Cardiac arrests (CA) is a leading cause of death worldwide [1, 2]. Although recent developments in cardiopulmonary resuscitation (CPR) techniques and post-resuscitation care have improved the chances of survival, there are still high rates of death and disability following the restoration of spontaneous circulation (ROSC), mainly due to cerebral injury [3–5].Survivors of CA suffer from painful sequelae, including anoxic brain injury, myocardial dysfunction and the systemic ischemia/reperfusion response, which are described as post cardiac arrest syndrome (PCAS). During the development of PCAS, the systemic ischemia/reperfusion response can trigger a systemic inflammatory cascade, which is the typical pathological process of CA and contributes to the forming of multiple organ dysfunction disease (MODS). This process is similar to the systemic inflammation response syndrome (SIRS), since pro-inflammatory factors such as TNF-α, IL-1βand NF-κB can be viewed as injury biomarkers in PCAS [6].The brain consumes the largest amount of oxygen of all organs, and is highly susceptible to disruptions of blood flow. Sudden cardiac arrest induces complete cerebral ischemia, followed by a cascade of detrimental events that can lead to immediate and delayed brain damage, including excitotoxicity, oxidative stress and inflammation [7, 8].Moreover, cardiopulmonary resuscitation can lead to reperfusion injury, which may exacerbate brain damage. Neurons in affected areas of the brain undergo delayed cell death, which disrupts the shaping of neural circuits and ultimately leads to both motor and cognitive dysfunction. Because of the high incidence of CA and the complex etiology of cerebral ischemia-reperfusion injury, it is urgent to find a therapeutic strategy to attenuate post-CA brain injury.

Dexmedetomidine (Dex) is a specific agonist of α2-adrenergic receptors that has been used as a sedative in intensive care since 1999, and also as an adjuvantto reduce the dosage of other anesthetics [9, 10]. Recently, a growing body of research found neuroprotective effects of Dex in different experimental models of cerebral injury [11–13]. While the uncontrolled, systemic inflammatory response is a critical cause of brain injury following ischemia/reperfusion (I/R), it was found that Dex can reduce the expression of pro-inflammatory factors after brain I/R injury, which may be related to the inhibition of the toll-like receptor-4//NF-κB (TLR-4/NF-κB) pathway [14, 15]. In addition, a recent randomized controlled trial indicated that Dex can lower the incidence of SIRS following percutaneous nephrolithotomy and reduce the expression of TNF-α and IL-1β [16]. Moreover, RCT research shows that Dex can attenuate the apoptosis of myocardial and renal cells [17]. However, whether these pharmacological effects of Dex could alleviate post-CA brain damage was not known, and the underlying mechanism have not been fully understood.

The present study used a rat model of CA/CPR to investigate the protective effects of Dex against brain injury as well as the potential mechanisms.

## Methods

### Animals and sample size

All the experiments were approved by the Animal Care and Use Committee of the Sichuan Academy of Medical Sciences and Sichuan Provincial People’s Hospital and the animals received humane care in compliance with the Guide for the Care and Use of Laboratory Animals published by the US National Institutes of Health (NIH Publication No. 85–23, revised 1996). A priori power analysis was carried out to determine the number of animals needed for our experiments. We used the on-line power analysis tool the G-Power. NDS was the primary evaluation index of the study. Based on the literature (Xu J,Zhonghua Wei Zhong Bing Ji Jiu Yi Xue.2020),we hypothesized that Dex increase the NDS by 10% compared to control, and the resulting number of animals was predicted to be 6 per group with an alfa error of 0.05 and a power of 0.8. The results of this *t*-test yielded an average group size of 6 animals and effect size (ES) > 1. Owing to approximately 50% survival rates after CA/CPR, our study finally enrolled 12 animals in the Control group and Dex group, respectively.

A total of 30 male Sprague-Dawley rats, weighing 350-450 g, aged 7 to 9 weeks, were obtained from the Chengdu Dashuo Experimental Animal Centre of Sichuan, China. The animals were housed at a constant temperature (23 ± 1 °C) on a 12 h light/dark cycle with free access to food and water, with two rats per cage. The housing environment was maintained until the animals were deeply anesthetized with 5% isoflurane for euthanasia and perfused transcardially with cold normal saline at 5 days after ROSC.

### Asphyxia cardiac arrest model

The asphyxia CA model in rat was established in the previous model [18], with minor modifications as follows. The rats were anesthetized with pentobarbital sodium solution (45 mg/kg) intraperitoneal injected and mechanically ventilated (respiratory frequency 60 bpm, tidal volume 8 ml/kg) using a Harvard Ventilator (Model 683, Harvard Apparatus, Holliston, MA, USA). The rats were lay on the back and the body temperature monitored by a rectal probe, which was maintained at 36 °C ± 1 °C with a heating pad. The right femoral artery were exposed and a venous indwelling catheter (24G) inserted, which was filled with heparin saline. To monitor the arterial blood pressure, the 24G venous was attached to a pressure transducer (Powerlab 16/30, AD-Instruments, Australia) and monitored for at least 10 min to record the baseline. Another 24G venous indwelling catheter was placed into the right femoral vein with saline. The asphyxia CA model, after muscle relaxation by cisatracurium besilate (0.2 mg/kg), was induced by clamping the tube in the trachea and stopping the ventilator. The systolic blood pressure (SBP) < 25 mmHg is the main indexes of CA and the condition? lasts for 6 min, after which CPR and mechanical ventilation were initiated. The chest compressions were performed on the 1/3 diameter of the rat thorax with a frequency of 200/min. During resuscitation, epinephrine (0.01 mg/kg), 5% sodium bicarbonate (0.36 mL /kg) and 0.9% normal saline (0.5 mL) were injected through the 24G venous in femoral vein. Resuscitation spontaneous circulatory recovery (ROSC) was defined at the restoration of spontaneous sinus rhythm and the retraction of > 60 mmHg for at least 10 min. Spontaneous respiration was carefully monitored every 5 min. The rats were removed from the ventilator after the complete recovery of spontaneous respiration. Finally, the intravenous indwelling catheters were extracted.

### Experimental protocol

All rats were randomly assigned to three groups: 1) sham operation group (sham, *n* = 6); 2) CA/CPR without any treatment (control, *n* = 12); 3) CA/CPR plus post-treatment with dexmedetomidine (Dex, *n* = 12). The tail of each rat was marked by different color markers according to group design, and the cage was labeled the group name. The sham rats went through all the operational procedures except for cardiac arrest and CPR. After restoration of spontaneous circulation, rats in the Dex and control groups received a single intravenous injection of Dex (25 mg/kg, Hengrui Medicine, Jiangsu, China) or the same volume of saline, respectively. Dex or saline was pumped into the vein using a micro-infusion pump for about 30 min.

### Evaluation of neurological deficits

Neurological examination was performed by an investigator who was blinded to the experimental design using the neurological deficit scores (NDS), which ranges from 80 (best) to 0 (brain dead) and includes a subscore of general behavioral deficit: consciousness as normal, stuporous or unresponsive and arousal with eye opening and respiration as normal, abnormal (hypo or hyperventilation) or absent. The NDS of the surviving rats was assessed at 24, 48 and 72 h after CA/CPR. The brainstem function sub-scores were assessed as follows: (1) olfaction, as response to the smell of food; (2) vision, as head movement toward light; presence of (3) pupillary light reflex; (4) corneal reflex; (5) startle reflex; (6) response to whisker stimulation and (7) swallowing of liquids or solids. The sub-score for motor assessment included strength testing as normal, abnormal (either stiff or weak) and absence of movement. The sensory assessment sub-score included response to limb pinch as brisk withdrawal, weak or abnormal response (extension or flexion posture) and no response. The motor behavior sub-score was assessed based on gait coordination as normal, abnormal or none. Balance on a beam was judged as normal if the rat could cross a 2 cm wide and 1 m long beam suspended 0.5 m above the floor; abnormal if the rat attempts and does not continue or stays momentarily and falls. The assessment was scored as absent if the rat falls off immediately upon placing on the beam. Other evaluated behavioral reflex sub-scores include: (1) righting reflex (animal placed on its back and is able to correct to upright position); (2) turning alley (the animal is made to walk and turn back at the end of a 15 cm by 0.5 m alley); (3) visual placing (the animal is lifted and is able to visually orient itself to objects and depth); and (4) negative geotaxis (animal is placed on its back on a plane angled at 45° and the animal corrects itself and moves upward on the incline). The last subscore assesses the occurrence of seizures (convulsive or non-convulsive).

### Rotarod test

The rotarod test is designed to evaluate the motor coordination and balance ability of rats. It includes adaptation training and a test process. Before CA surgery, the rats in each group were trained continuously for 3 days. The rotating bar fatigue meter was set to 4 rpm. The animals were trained 3 times a day for at least 15 min each time, and the interval between the two training sessions was at least 15 min. The final testing was performed 5 days after ROSC. All surviving rats were individually placed on the rotating rod, the rotation speed was increased from 4 rpm to 40 rpm within 260 s and the time from the beginning to the fall of the rat recorded. The test was repeated three times, and the average amount of time until falling was taken as the finally result.

### Serum levels of inflammatory factors

Retro-orbital blood samples (0.8–1.2 mL) were collected at 2, 24, and 48 h after ROSC, the serum was separated by centrifugation at 12000 g for 10 min and immediately analyzed or stored at − 80 °C. The levels of IL-1βand TNF-α were analyzed using commercial ELISA kits (R&D systems) according to the manufacturer’s instructions. All measurements were carried out in duplicate.

### Western blot analysis

Bilateral Frontal cortices anterior to the bregma were dissected at 5 days after ROSC, and the left cortical tissues were homogenized for western blotting. Total protein lysates were prepared using lysis buffer (Thermo Scientific, Rockford, IL, USA) containing protease inhibitors cocktail (Sigma-Aldrich) and phosSTOP phosphatase Inhibitor Cocktail (Roche, Nutley, NJ, USA). The BCA assay kit (Thermo Fisher Scientific, USA) was used to measure the protein concentration. Samples comprising 20 μg total protein per lane was separated by SDS-PAGE and then transferred to a PVDF membrane. The membranes were blocked with 5% non-fat milk for about 1 h at room temperature and incubated with the following primary antibodies overnight at 4 °C: rabbitpolyclonal anti-caspase-3 antibody (1:1000; Cell Signaling Technology, USA); rabbit polyclonal anti-NF-κB antibody (1:1000Protein-tech, China);α-tubulin (1:5000; Protein-tech, China,). After incubation with secondary antibodies, the immunoreactive bands were developed using enhanced chemiluminescence reagents (Pierce, IL, USA) and was visualized using GeneSnap software version 7.08. The protein amounts were quantified using Image J software and normalized to the density of α-tubulin in the same sample. The results of rats from the different experimental groups were then normalized to the mean values of the corresponding control animals.

### TUNEL staining

The TUNEL assay was performed using the Apoptosis & Cell Death Assay kit (Merck Millipore, USA) according to the manufacturer’s instructions. Briefly, right frontal cerebral cortical tissue sections were incubated with proteinase K at room temperature for 30 min, and then incubated with the TUNEL reagent at 37°Cfor 1 h. The sections were then washed with PBS and counter-stained with 4′,6-diamidino-2-phenylindole (DAPI). Fluorescence images were captured using a fluorescence microscope at 40 × magnification. The results were quantified as apoptotic index (AI%), which was defined as the ratio of positive apoptotic cells to all cells in the same field of view.

### Statistical analysis

The results are presented as the means ± SD. (*n* ≥ 6. Repeated measures or samples analysis of variance, as appropriate, was performed to compare two or more groups. All pairwise comparisons were carried out with pairwise *t*-tests. Survival curves were determined using the Kaplan-Meier method and compared using the log-rank test. Differences with *p*-values of less than 0.05 were considered statistically significant. Statistical analyses were performed using SPSS 20.0 software (IBM Corp., USA) and charts were rendered using Prism 6.0 software (GraphPad Software Inc., USA).

## Results

### Dex postconditioning reduces MAP and HR but does not affect the survival rate after CA/CPR

**Fig. 1:**
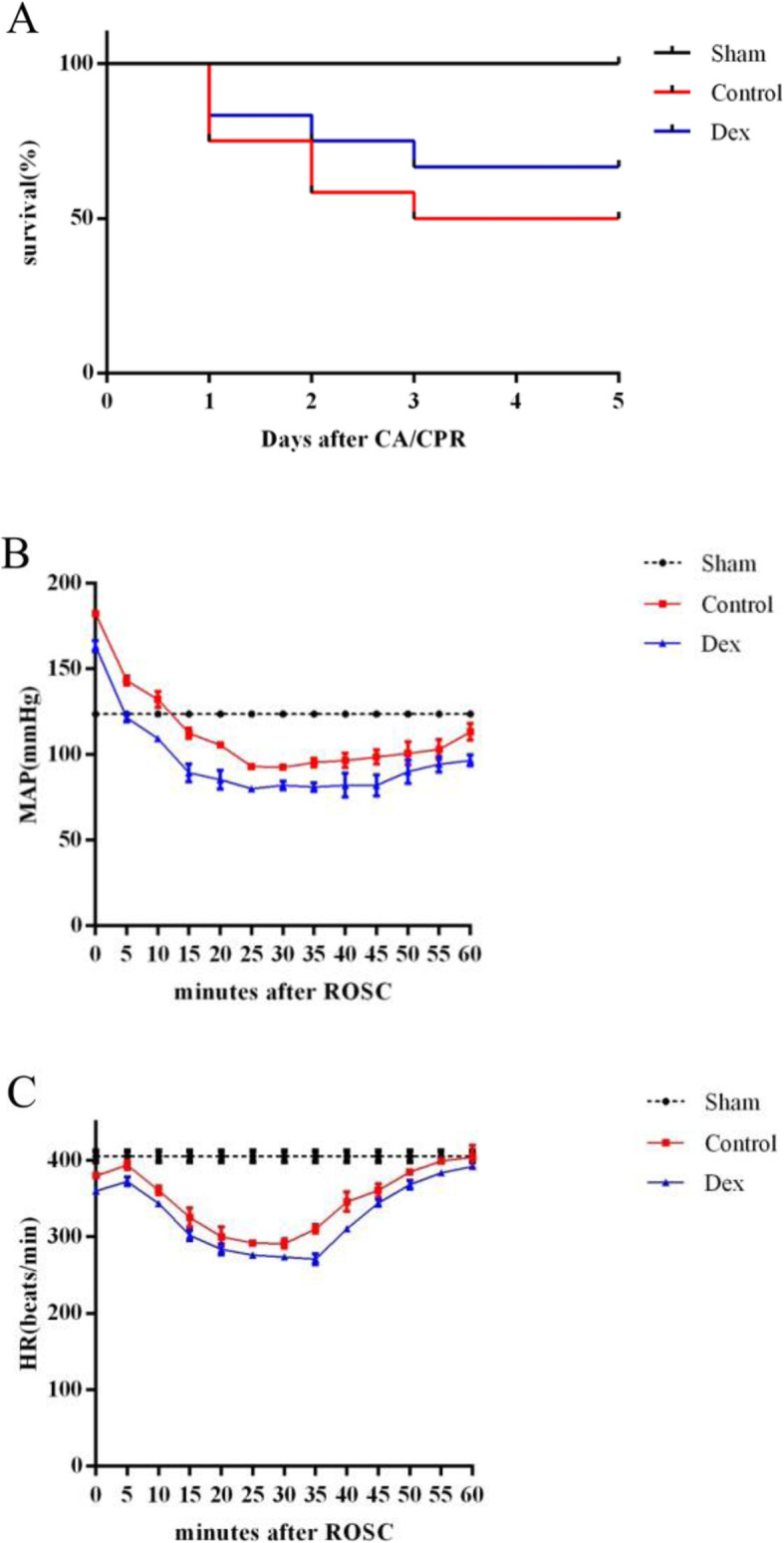
Dex improved the survival and hemodynamics of rats following cardiac arrest and cardiopulmonary resuscitation (CA/CPR). **A** Survival of the Sham, Control and Dex groups. **B** and **C** Changes in the mean arterial pressures (MAP) and heart rate (HR) in the Control and Dex groups after CA/CPR. Data are expressed as the means ± SD (*n* = 6–12). *P < 0.05

All six rats in the Sham group survived. The survival rates of the rats in the Control and Dex groups were 50 and 66.7%, respectively. Data on six animals in the control group and eight animals in the Dex group were used for analysis. Consistent with the survival rates assessed at 7 days after CA/CPR, there were no significant difference between the control group and the Dex group (*p* > 0.05) (Fig. 1a). Compared with the basal level, the MAPs of the control and Dex groups decreased significantly after resuscitation, and the most notable decreases were observed at 15–25 min after ROSC (*P* < 0.05). The reduction of blood pressure was lower in Dex group than in the control group (*p* < 0.05) (Fig. 1b and Table 1). Similarly, compared with the basal values, the heart rate (HR) decreased in both groups (*p* < 0.05). The reduction in the Dex group was greater than in the control group, but both returned to the baseline 1 h after ROSC (Fig. 1c and Table 1). These results suggest that Dex postconditioning reduces hemodynamic change, but does not decrease the survival rate.

**Table 1:** The change of MAP and HR after CA/CPR^a,b^

Minutes after CA/CPR(min)	MAP(mmHg)			*p*-value	HR(beats/min)			*p*-value
	Sham (*n* = 6)	Control (*n* = 12)	Dex (*n* = 12)		Sham (n = 6)	Control (*n* = 12)	Dex (*n* = 12)	
0	125.17 ± 2.79	180.92 ± 4.74	162.42 ± 5.45	< 0.0001	416.50 ± 3.83	386.67 ± 2.10	371.00 ± 1.35	< 0.0001
5	125.17 ± 2.79	145.92 ± 3.32	124.08 ± 2.57	< 0.0001	415.00 ± 4.15	397.08 ± 1.83	380.75 ± 1.42	< 0.0001
10	125.17 ± 2.79	135.42 ± 2.68	112.50 ± 1.78	< 0.0001	412.83 ± 4.22	371.92 ± 1.51	360.08 ± 1.73	< 0.0001
15	125.17 ± 1.94	116.33 ± 1.50	91.42 ± 4.58	< 0.0001	415.33 ± 3.83	348.67 ± 3.20	323.58 ± 3.73	< 0.0001
20	124.67 ± 1.51	106.17 ± 1.53	85.25 ± 3.19	< 0.0001	415.00 ± 2.45	329.33 ± 2.77	295.75 ± 1.66	< 0.0001
25	125.67 ± 1.97	100.75 ± 1.42	78.00 ± 1.13	< 0.0001	413.83 ± 2.40	300.00 ± 2.30	285.75 ± 2.22	< 0.0001
30	125.50 ± 2.59	102.75 ± 1.14	82.00 ± 0.95	< 0.0001	414.67 ± 1.97	300.08 ± 2.15	280.25 ± 1.60	< 0.0001
35	125.50 ± 1.38	105.08 ± 0.79	80.42 ± 1.00	< 0.0001	415.33 ± 2.73	312.42 ± 1.78	277.33 ± 1.61	< 0.0001
40	124.67 ± 2.73	107.33 ± 1.50	84.58 ± 4.54	< 0.0001	415.00 ± 3.69	346.50 ± 5.66	309.75 ± 1.71	< 0.0001
45	125.67 ± 1.51	108.25 ± 0.97	84.67 ± 4.60	< 0.0001	415.50 ± 3.94	361.25 ± 10.04	345.00 ± 3.16	< 0.0001
50	125.67 ± 2.42	111.08 ± 1.83	94.67 ± 6.10	< 0.0001	415.50 ± 4.46	383.92 ± 2.39	359.25 ± 8.02	< 0.0001
55	126.17 ± 2.79	114.50 ± 1.45	100.00 ± 3.30	< 0.0001	414.67 ± 4.03	407.42 ± 1.83	385.00 ± 2.89	< 0.0001
60	125.83 ± 1.94	120.08 ± 2.02	105.58 ± 1.08	< 0.0001	415.33 ± 4.08	417.17 ± 5.62	396.42 ± 3.58	< 0.0001

^a^ Data are mean ± SD

^b^ *p*-value<0.05

Table 1Changes in the mean arterial pressures (MAP) and heart rate (HR) in the Control and Dex groups after CA/CPR (*n* = 6–12)

### Dex attenuated the impairment of neurocognitive function, motor coordination and balance following CA/CPR

**Fig. 2:**
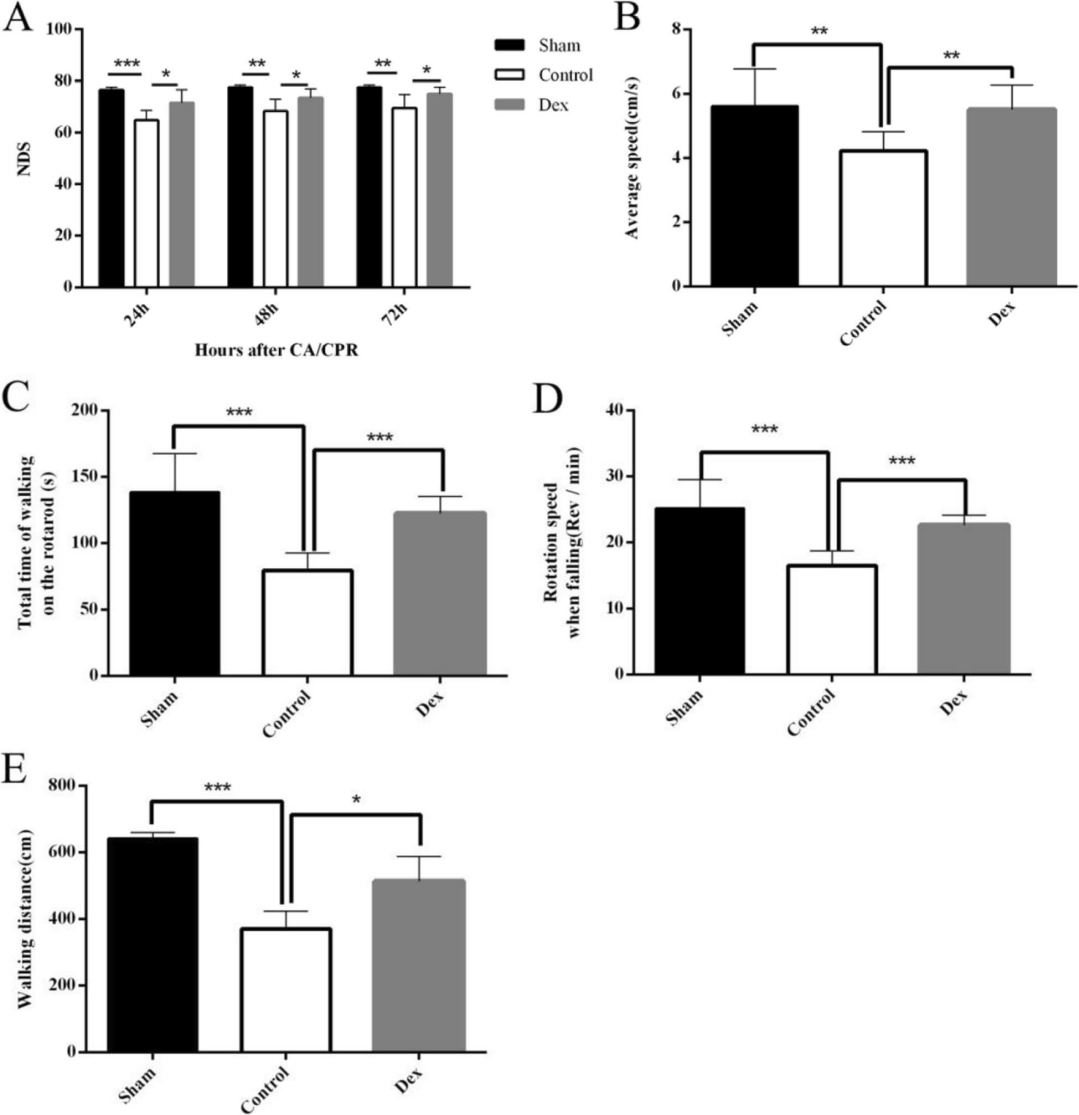
Dex attenuated the impairment of neurological and motor function after CA/CPR. **A** The neurological deficit score evaluated at24, 48 and 72 h after ROSC. **B**–**D** Rotarod performance tests were conducted at 5 days after ROSC. The results are shown as the average speed of the rotarod (**B**), the total time of walking on the rotarod (**C**), the rotation speed at falling (**D**) and the total distance of rat the walking on the rotarod (E). The data are presented as the means ± SD (*n* = 6). ***p < 0.001; **p < 0.01; *p < 0.05

The NDS was evaluated at 24, 48 and 72 h after CA/CPR. In the sham group, the NDS score was approximately 80 at all the time points. After CA, the NDS of the control group was obviously decreased compared with the sham group. Treatment with Dex markedly attenuated the neurological deficit score (Fig. 2a).

In addition to neurological function disorders, CA/CPR can also damage motor function. In this study,the rotarod test was used to assess the ataxia of rats at 5 days after CA. After CA/CPR, the surviving rats in the Control group showed significantly poorer scores in all indicators, including the average rotarod speed (Fig. 2b), the total time on the rotarod (Fig. 2c), rotation speed at fall (Fig. 2d) and the total walking distance (Fig. 2e). As can be seen in the corresponding figures, the application of Dex effectively attenuated the neurological impairment.

### Dex reduced the expression of pro-inflammatoryfactors following CA/CPR

**Fig. 3:**
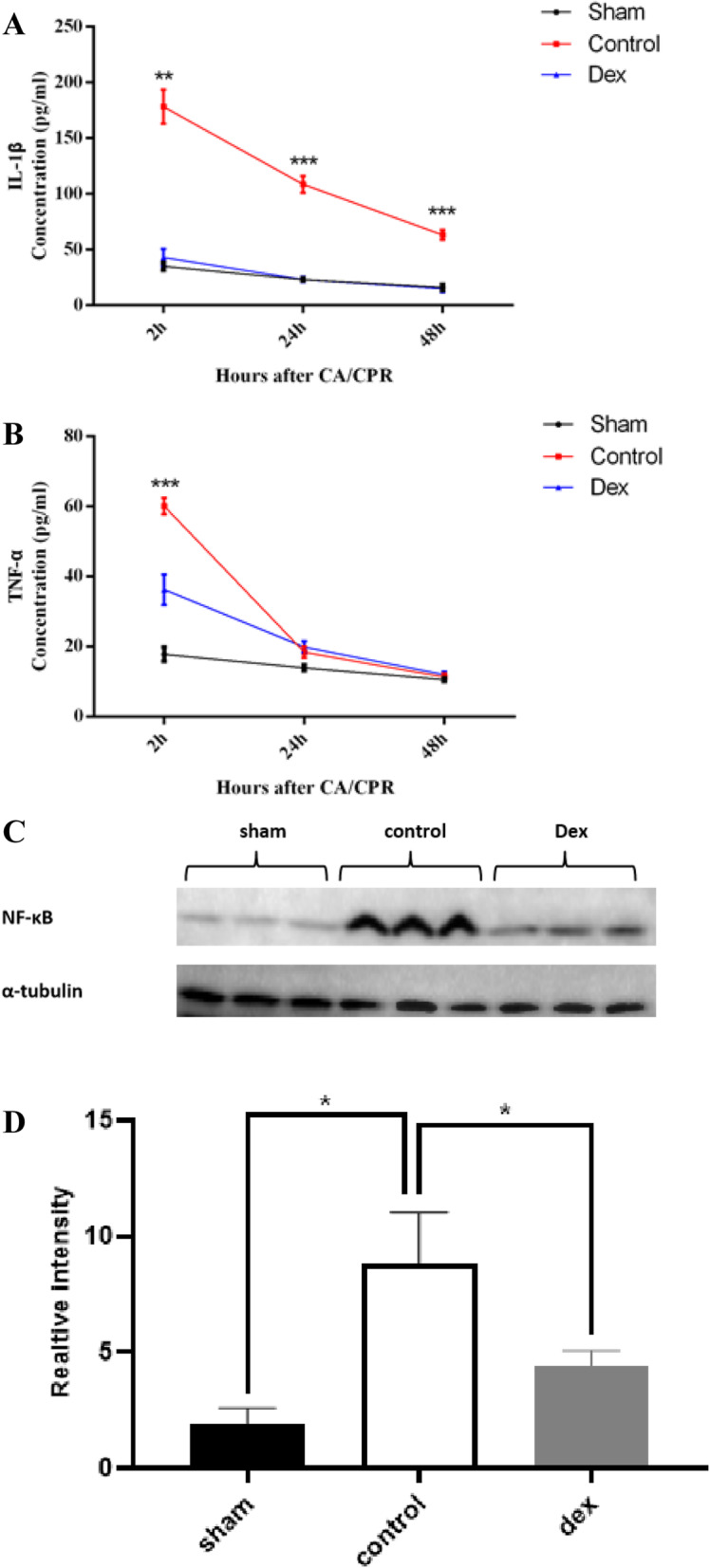
Dex reduced the expression of pro-inflammatory cytokines after CA/CPR. The serum levels of IL-1β (**A**) and TNF-α (**B**) at 2, 24 and 48 h after resuscitation. **C**–**D** The protein levels of NF-κB in brain tissues. The data are presented as the means ± SD (*n* = 6). *p < 0.05

To evaluate the anti-inflammatory effect of Dex following CA/CPR operation, the serum levels of IL-1β and TNF-α, as well as the expression of NF-κB in brain tissues were evaluated at 2, 24 and 48 h after CA/CPR. Compared with the sham group, the serum levels of IL-1β and TNF-α (Figs. 3a and b) and the brain tissue expression of NF-κB (Fig. 3c) were significantly increased following CA/CPR. However, application of Dex after resuscitation decreased the production of IL-1β and TNF-α, while also blocking the increase of NF-κB in the brain (Fig. 3).

### Dex inhibited the expression of proteins related to neuronal apoptosis following CA/CPR

**Fig. 4:**
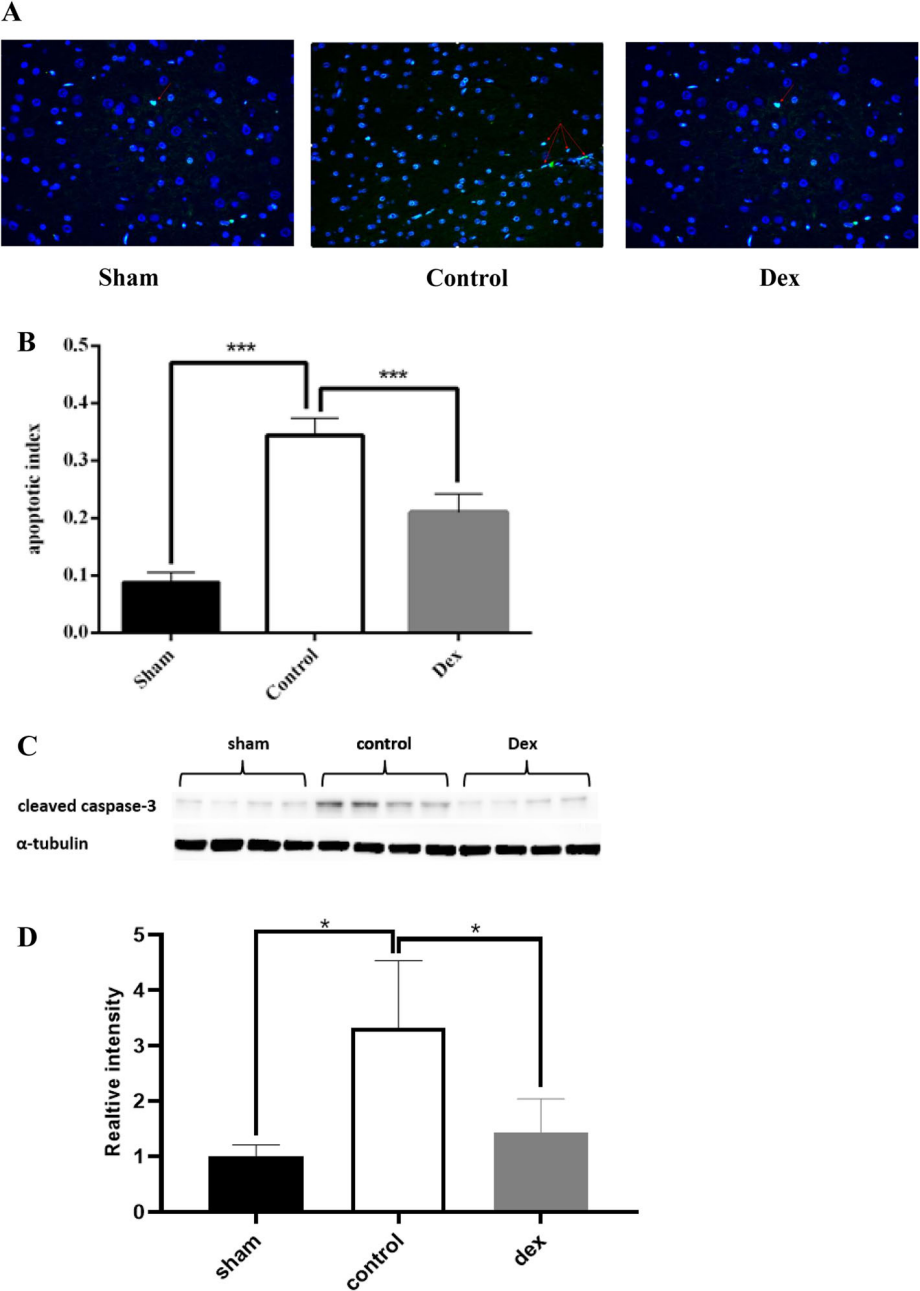
Dex inhibits the apoptosis of neurons after CA/CPR. CA/CPR-induced apoptosis, as assessed by TUNEL staining. **A** The quantitative analysis results of TUNEL staining. **B** Treatment with Dex decreased the levels of cleaved caspase-3. **D** Western blot analysis results, normalized to α-tubulin. The data are expressed as the means ± SD and the scale bar of TUNEL staining is 100 μM (*n* = 4–6).*P < 0.05

To understand whether the protective effect of Dex is related to neuronal apoptosis, TUNEL staining and western blot analyses were performed to assess the percentage of apoptotic neurons and expression of the proapoptotic factor caspase-3. According to TUNEL staining, there were few positive cells in the sham group, but their percentage increased after CA/CPR (Fig. 4a). Furthermore, Dex significantly decreased the number of TUNEL-positive neurons (Fig. 4a). The proportion of TUNEL-positive neurons was done by cell counting in a single field of view, which showed that the apoptosis index of CA/CPR rats was significantly increased, while Dex effectively blocked this increase (Fig. 4b). In addition, treatment with Dex significantly reduced the expression of caspase-3 (Fig. 4c and d).

## Discussion

In this study, we explored the potential effects of Dex on the survival and neurological function of rats after CA/CPR. The main findings of this study include: 1) Dex postconditioning reduced the MAP and HR at 1 h after successful resuscitation but did not affect the 5-daysurvival rate after CA/CPR. 2) Dex ameliorated the CA/CPR-induced neurological deficits; and 3) Dex may exert its protective effect by reducing inflammation and inhibiting apoptosis.

CA/CPR induces systemic ischemia-reperfusion (I/R) injury, which activates the immune system and causes a systemic inflammatory response. During CA/CPR, leukocytes, macrophages and tissue-resident immune cells recognize the injury and release primary cytokines, which in turn induce the recruitment and activation of leukocytes, largely amplifying the inflammatory response [19, 20]. The brain uses 20% of the body’s oxygen and calories [21], and can therefore suffer severe damage due to CA. As the crucial resident immune cells of the central nervous system (CNS), microglia express various cytokine receptors, recognizing IL-1 and TNF-α, among many others. Consequently, microglia will be over-activated after I/R injury and release excess pro-inflammatory cytokines, impairing neural function [22, 23]. A growing body of evidence suggests that inflammation is crucial for the pathogenesis of neurological deficits after CA/CPR [24–27]. In this study, we found that the serum levels of TNF-α andIL-1βwere significantly increased after ROSC in the CPR group. The levels of pro-inflammatory factors peaked at 2 h and returned to baseline levels within 48 h after ROSC. Treatment with Dex attenuated the increase of TNF-α and IL-1β,improving the neurological outcomes. CA/CPR can cause sympathetic nerve over-excitation, which may exacerbate further inflammation and cause significant neurotoxicity [28, 29]. Dex is a highly specific agonist of the α2-adrenergic receptor and is commonly used as an adjuvant anesthetic. Furthermore, several studies demonstrated the anti-inflammatory effect of Dex in different models. For instance, Dex was found to significantly improve cognitive function after carotid endarterectomy by inhibiting CNS inflammation [30]. Moreover, Zheng and colleagues showed that Dex inhibited CNS neuroinflammation after traumatic brain injury (TBI), and reduced the expression of the nucleotide-binding oligomerization domain (NOD)-like receptor family pyrin domain containing 3 (NLRP3) inflammasome [31]. Additionally, previous research suggests that post-treatment with Dex could attenuate early brain injury (EBI) induced by subarachnoid hemorrhage (SAH), and that it exerts its protective effect by inhibiting the activation of the TLR4/NF-κB pathway, the release of pro-inflammatory cytokines and the expression of the NLRP3 inflammasome [32]. NF-κB is a transcription factor that regulates many genes, especially inflammation-related cytokines. Dex was shown to reduce the expression of Toll-like receptor 4 and suppress the activation of NF-κB by interacting with the α-2 receptor [33]. The results of this study are agreement with this theory, and we found that treatment with Dex can effectively suppress the phosphorylation of NF-κB following CA/CPR.

Recently, there has been increasing evidence that neuronal apoptosis is also a key reason for CNS dysfunction after I/R [5, 34]. Apoptotic programmed cell death is mainly induced by specific proteins such as Apaf-1, as well as proteins in the Bcl-2 and caspase families [35]. In mammalian cells, apoptosis is triggered by two main pathways, called the intrinsic pathway and the extrinsic pathway, which both converge in the activation of caspase-3 [36, 37]. Neuronal apoptosis is initiated by the cleavage of caspase-3 and results in DNA breakdown, degradation of cytoskeletal components, and the production of apoptotic particles, which are finally engulfed by phagocytic cells [38]. CA/CPR compromises the integrity of the blood-brain barrier and activates microglial cells [39], resulting in the release of inflammatory mediators and reactive oxygen species. These toxic chemicals inhibit the production of neurotrophic factors and disturb the effective communication between brain cells [40]. In this study, TUNNEL staining showed a significant increase in the number of apoptotic neurons following CA/CPR, which could be effectively alleviated by Dex. The level of cleaved caspase-3 is universally recognized as a specific marker of apoptosis [17]. Our findings indicated that Dex decreased the concentration of cleaved caspase-3 in brain tissues, which was in accordance with the results of TUNEL-staining. Previous research demonstrated that Dex exerts its antiapoptotic effect by reducing the levels of pro-inflammatory factors and ROS [41, 42]. In addition, Dex improves the survival of neurons by activating the brain-derived neurotrophic factor/ tropomyosin-related kinase B(BDNF/TrkB)pathway [43, 44].

The current study also has some inadequacies and limitations. For example, we were not able to use a concentration gradient to confirm the best therapeutic dose due to limitations in the number of experimental animals that can be handled. What’s more, in additon, exploration of the possibility of combination therapy, such as dexmedetomidine combined with hypothermia therapy, is still awaiting further research.

## Conclusions

Our findings indicate that post-resuscitation treatment with dexmedetomidine has a significant neuroprotective effect and attenuates neurological disorders following CA/CPR. The potential mechanism through which dexmedetomidine exerts its protective effects is likely related to the suppression of neuroinflammation and promotion of neuron survival by inhibiting apoptosis.

## Data Availability

All data generated or analyzed during this study are included in this published article and supporting data can be obtained from the corresponding author upon reasonable request.

## Abbreviations

AI: Apoptotic index
BDNF: Brain-derived neurotrophic factor
CA: Cardiac arrest
CNS: Central nervous system
CPR: Cardiopulmonary resuscitation
DAPI: 4′,6-diamidino-2-phenylindole
Dex: Dexmedetomidine
EBI: Early brain injury
HR: Heart rate
IL: Interleukin
I/R: Ischemia reperfusion
MAPs: Mean arterial pressure
NDS: Neurological deficit scores
NF-κB: Nuclear factor-kappa B
NIH: National Institutes of Health
NOD: Nucleotide-binding oligomerization domain
NRLP3: (NOD)-like receptor family pyrin domain containing 3
ROSC: Restoration of spontaneous circulation
SBP: Systolic blood pressure
SAH: Subarachnoid hemorrhage
TBI: Traumatic brain injury
TNF-α: Tumor necrosis factor alpha
TrkB: Tropomyosin-related kinase B
TLR-4: Toll-like receptor 4
